# Antiangiogenic Effect of Alkaloids

**DOI:** 10.1155/2019/9475908

**Published:** 2019-04-21

**Authors:** Masoud Alasvand, Vahideh Assadollahi, Roberto Ambra, Ehsan Hedayati, Wesam Kooti, Ilaria Peluso

**Affiliations:** ^1^Department of Medical Physiology and Pharmacology, Faculty of Medicine, Kurdistan University of Medical Sciences, Sanandaj, Iran; ^2^Cellular and Molecular Research Center, Research Institute for Health Development, Kurdistan University of Medical Sciences, Sanandaj, Iran; ^3^Council for Agricultural Research and Economics, Research Centre for Food and Nutrition (CREA-AN), Via Ardeatina 546, 00178 Rome, Italy; ^4^Student Research Committee, Ahvaz Jundishapur University of Medical Sciences, Ahvaz, Iran; ^5^Cellular and Molecular Research Center, Sabzevar University of Medical Sciences, Sabzevar, Iran

## Abstract

Alkaloids are among the natural phytochemicals contained in functional foods and nutraceuticals and have been suggested for the prevention and/or management of oxidative stress and inflammation-mediated diseases. In this review, we aimed to describe the effects of alkaloids in angiogenesis, the process playing a crucial role in tumor growth and invasion, whereby new vessels form. Antiangiogenic compounds including herbal ingredients, nonherbal alkaloids, and microRNAs can be used for the control and treatment of cancers. Several lines of evidence indicate that alkaloid-rich plants have several interesting features that effectively inhibit angiogenesis. In this review, we present valuable data on commonly used alkaloid substances as potential angiogenic inhibitors. Different herbal and nonherbal ingredients, introduced as antiangiogenesis agents, and their role in angiogenesis-dependent diseases are reviewed. Studies indicate that angiogenesis suppression is exerted through several mechanisms; however, further investigations are required to elucidate their precise molecular and cellular mechanisms, as well as potential side effects.

## 1. Introduction

Alkaloids are among the natural phytochemicals contained in functional foods and nutraceuticals [1] and have been suggested for the prevention and/or management of oxidative stress and inflammation-mediated diseases [1, 2]. In this review, we aimed to describe the effect of alkaloids on angiogenic vessel formation from a previous existing capillary, a process that is implicated in many physiological conditions such as wound healing and menstrual cycle and pathological conditions such as tumor growth or retinopathy [3–5].

The angiogenic process is a cascade of events resulting in new lumen-containing vessels, through the dissolution of the vascular basal membrane, migration of endothelial cells from the parent vessel toward developing blood vessels, and widespread cellular proliferation, which are activated by several proangiogenic factors. When blood flow is initiated, physiological processes such as embryonic development, wound healing, and immune reactions are then allowed to start and develop [6, 7]. On the other hand, the creation of new blood vessels increases the supply of nutrients, oxygen, and growth factors to normal and tumor cells. If tumor cells can induce angiogenesis, subsequent tumor expansion and transition from a benign state to a malignant one is started [8]. Endothelial cells can migrate in order to initiate or progress to angiogenesis, thanks to a wide range of regulators and signaling molecules such as basic fibroblast growth factor (bFGF), epidermal growth factor (EGF), vascular endothelial growth factor (VEGF), hepatocyte growth factor (HGF), and transforming growth factor (TGF). Thus, these angiogenic peptides play a critical role in the creation of new blood vessels [9–11]. In both physiological and pathological conditions, HIF-1 is a critical mediator of hypoxic response and O_2_ homeostasis as well as an essential angiogenic regulator. Hypoxia leads to HIF-1*α* stabilization, a subunit of the heterodimeric transcription factor HIF-1, and increased VEGF production [12, 13]. Oxygen demand can be increased by excessive cell proliferation during embryonic development and/or tumor growth [14].

Moreover, VEGF is the main factor that initiates angiogenesis. Similar to some proangiogenic genes, VEGF also has hypoxia response element (HRES) as the binding site of HIF-1 within the promoters. In hypoxic conditions, HIF-1 directly stimulates VEGF and its receptors, inducing angiogenesis [15, 16]. The abovementioned mechanisms in the formation of new blood vessels demonstrate that VEGF directly links hypoxia with angiogenesis initiation. The VEGF family of growth factors exerts its effects by interacting with receptor tyrosine kinases (RTKs) named vascular endothelial growth factor receptors (VEGFRs). Activation of this signaling pathway leads to endothelial cell (EC) proliferation, extracellular matrix degradation, EC migration, and then new blood vessel formation [17, 18]. Understanding the tumor angiogenesis signaling pathways is an important treatment goal of malignancy and cancer therapy. However, because of the similarities between tumor and physiologic angiogenesis signaling pathways, insufficient efficacy and resistance may be challenges we face during such cancer therapy [19].

The results of different studies show that several molecular pathways such as VEGFRs, Ephrin-Eph receptors, and the Delta-like ligand and neurogenic locus notch homolog protein (Delta-Notch) system are involved in angiogenesis [7, 20].

## 2. Angiogenesis Signaling Pathways

VEGF belongs to the platelet-derived growth factor (PDGF) family; moreover, the VEGF family has five members in mammals, including VEGF-A, VEGF-B, VEGF-C, VEGF-D, and PlGF (placental growth factor). All of them stimulate cellular responses and are characterized by the presence of eight conserved cysteine residues forming the typical cysteine-knot structure [21]. VEGFRs are tyrosine kinase receptors (TKRs) with a cytoplasmic domain and tyrosine kinase activity, containing 3 to 4 members. VEGF-A, the most important member of this family, was obtained from tumor cells for the first time. VEGF-A regulates angiogenesis and vascular permeability via activation of VEGFR-1 and VEGFR-2. The binding of VEGF-A to its receptor represents the most important signal for angiogenesis. On the other hand, VEGF-C/VEGF-D and their receptor, VEGFR-3, are involved in lymphangiogenesis [22, 23]. Several VEGF family ligands and receptors, especially VEGF-A, are regulated by HIF; therefore, angiogenesis is intensified during healthy tissue growth and also in cancerous conditions [24]. Following the binding of VEGF-A to VEGFR2, the RAS/RAF (rapidly accelerated fibrosarcoma)/ERK (extracellular signal-regulated kinases)/MAPK (mitogen-activated protein kinases) pathway is activated potentially via sphingosine kinase (SPK) activated by protein kinase C (PKC). RAS target genes can now be enabled, inducing proliferation [25]. Recent studies consider the role of the transcription factor *nuclear factor-kappa beta* (NF-*κ*B), which interacts with RAS, in tumor angiogenesis; therefore, it can be an antiangiogenic therapeutic target. It has been demonstrated that oncogenic RAS mediates the activation of the NF-*κ*B pathway and subsequent upregulation of NF-*κ*B target genes, in particular the proangiogenic IL-8 cytokine, leading to lung tumorigenesis [26].

Other RAS-independent molecular pathways, such as the phospholipase C gamma- (PLC*γ*-) protein kinase C- (PKC-) MAPK pathway, are also implicated in VEGFR2 signaling [22]. Binding of VEGFR2 to VEGF-A and phosphorylation of several tyrosine residues in the cytoplasmic domain of this receptor can induce the activation of phospholipase C gamma (PLC*γ*). On the one hand, PLC*γ* releases diacylglycerol (DAG) which recruits PKC to the cell membrane and promotes cell proliferation; on the other hand, PLC*γ*-mediated Ca^2+^ mobilization leads to increased vascular permeability, promoting leakage of plasma proteins into the extravascular space, resulting in a fibrin gel formation marking the proper temporary place for the placement of the new blood vessel, and supporting its growth [23, 27, 28].

Generally, the phosphorylation of different tyrosines in the cytoplasmic domain of VEGFR2 and the subsequent recruitment of signal transducers promote migration and cell mobility and regulate cell attachment, all of which are needed for angiogenesis [29]. As already mentioned, different molecular pathways are involved in pathophysiological angiogenesis compared to the physiological one [30]. The phosphatidylinositol 3-kinase (PI3K)/AKT/mammalian target of rapamycin (mTOR) pathway is activated in many human cancers. The activation of the PI3K/AKT pathway in cancer cells can increase VEGF production. This may be done through HIF-1-dependent mechanisms, or it may occur in an HIF-1-independent manner. In tumor cells, PI3K/AKT activation can occur because of RAS mutation which is responsible for tumor cell feeding [12]. Other signaling pathways, such as JAK-STAT, can promote the migration of endothelial cells and subsequent tumor angiogenesis [31].

On the other hand, VEGFR1 is a negative regulator of angiogenesis. VEGFR1 is a decoy receptor, and the binding of VEGF-A to it may prevent the activation of VEGFR2. Also, Delta-like ligand 4 (DII4), a transmembrane ligand of notch family receptors, is a negative regulator of angiogenesis. More precisely, when VEGF binds to VEGFR2, it causes DII4 upregulation which in turn interacts with NOTCH1 receptors on adjacent endothelial cells, resulting in VEGFR2 downregulation in those cells. Finally, the activation of Notch signaling leads to a reduced number of tip cells and less dense vascular network formation via the prevention of tubular sprouting in angiogenesis [22, 32].

The involvement of coagulation factors in tumor-induced and normal angiogenesis has been reported in several studies [33, 34], supporting their role in tumor progression.

## 3. Angiogenesis Inhibitors and Cancer Treatment

Angiogenesis is the creation of new capillaries from preexisting vessels and circulating endothelial precursors [35]. It can be activated in physiological and pathological conditions, for example, in response to hypoxia, cancer, diabetic retinopathy, and inflammatory, infectious, and immune disorders [36]. Proangiogenic growth factors commonly switch on a series of surface receptors in a series of paracrine and autocrine circles with the VEGF-A signaling representing the critical rate-limiting step, physiologically and pathologically. VEGF-A is the main VEGF that binds to VEGFR2 to mediate vascular permeability, endothelial proliferation, migration, and survival [37].

Angiogenesis also plays a critical role in the growth of cancer for the reason that cancer tissues need a blood supply. Tumors can stimulate normal cells to generate angiogenesis signaling molecules. Angiogenesis inhibitors act through several ways. Some of them are monoclonal antibodies that recognize and bind to VEGF. When VEGF is attached to these drugs, it is unable to activate the VEGF receptor [38].

Other inhibitors bind to the VEGF receptor on the surface of endothelial cells or to other proteins in the downstream signaling pathways and block their activities. Some of the inhibitors are immunomodulatory drug agents that stimulate or suppress the immune system. One of the ideas of antiangiogenic therapy is through the destruction tumor vessels [39].

Since angiogenesis is the main requisite for cancer growth and invasion, its inhibition is considered the mainstay in cancer treatment strategies. The study of angiogenesis led to the identification of numerous proangiogenic factors as well as angiogenic suppressor proteins. By the identification of these proteins, a wide spectrum of angiogenesis inhibitors were introduced in cancer treatment procedures. Several antiangiogenic molecules currently in use employ anti-VEGF-A antibodies [40, 41] and tyrosine kinase inhibitors [42–45]. However, serious adverse effects have been reported for such molecules [46]. On the other hand, herbal drugs, most commonly alkaloid-based plants, are among the more promising angiogenic inhibitors to be used for the treatment of different types of cancers like prostate cancer cells, breast cancer cells, melanoma cells, and cervical cancer cells [47–51].

## 4. Role of MicroRNAs in Angiogenesis Regulation

The identification of proteins involved in angiogenic processes led to the identification of microRNAs (miRNAs) as potent inhibitors of angiogenesis and cancer. MiRNAs are single-stranded short noncoding RNA molecules (about 21-25 nucleotides) that modulate cellular target genes at the posttranscriptional level. These RNA molecules have a role in almost all biological processes and their potential role in most pathogenesis has been observed. Cancers and thrombotic and bleeding disorders, as well as heart disease, are among those disorders in which the potential role of miRNAs has been investigated. Defects in miRNA synthesis led to a wide spectrum of early disorders including impaired T-cell formation, cardiomyopathy, disrupted blood circulation, and impaired angiogenesis [52–55]. One of the most important consequences of the impaired biogenesis of miRNAs is early embryonic lethality due to defective vessel formation and maintenance [56–58]. These observations encouraged studies to evaluate the role of different miRNAs in angiogenic processes, and several miRNAs were identified with a potential role in the regulation of different aspects of angiogenesis.

It has been reported that some of the miRNAs, such as miR-126, miR-221/222, miR-17-92 cluster, miR-93, let-7f, and miR-214, modulate the response of the vascular endothelium to angiogenic stimuli and can be proangiogenic or antiangiogenic [59–62].

MiR-221 and miR-222 are two relatively well-known miRNAs with an important effect on protooncogene c-Kit (CD117) as a key role in the *survival* of hematopoietic stem cells. Further studies on endothelial cells revealed that these two miRNAs have antiangiogenic activity [63, 64]. Overexpression of miR-221 and miR-222 in endothelial cells led to increased endothelial nitric oxide synthase, which in turn resulted in the suppression of endothelial survival and angiogenesis. MiR-34a is another miRNA with potential antiangiogenic activity, as it was shown to induce endothelial progenitor cell (EPC) senescence and dysfunction. EPCs have a crucial role in angiogenesis and are important for many physiological processes, even pathological ones, such as the growth of tumor cells [65, 66]. MiR-34a suppresses silent information regulator 1 (Sirt1) resulting in senescence induction in EPCs [67]. Mir-126 is another miRNA with direct and indirect effects on angiogenesis that is mainly found in tissues with high vascular components, such as the lungs and the heart [68–70]. MiR-126 regulates many endothelial cell functions and processes mainly including cell migration and cell survival. MiR-93 is involved with the stimulation of angiogenesis and the promotion of tumor growth, and its inhibition is suggested as a tool for the suppression of angiogenesis and tumor growth [71, 72].

Endothelial cells express VEGF receptor genes. miRNAs can modulate angiogenesis by regulating VEGF receptor expression in endothelial cells, whereupon they influence the migration and invasion of cancer cells. In addition, recent studies showed that Dicer and Drosha, two enzymes that control the processing of miRNAs, play key roles in the angiogenic process. Not only do miRNAs modulate mRNA translation level, but mRNA also regulates the function of miRNAs. Thus, researchers can target the molecular pathways, taking part in the development of diseases, by determining miRNAs which are important in the angiogenic process [57, 59, 73, 74].

The number of known miRNAs with a direct or indirect role in angiogenesis is on the rise, and a summary is shown in Table 1.

## 5. Plant-Based Alkaloids

Nowadays, a wide variety of plants that are rich in alkaloids have been discovered and these compounds have a significant impact on angiogenesis-dependent diseases. Alkaloids have an antiangiogenic activity and act through several mechanisms inhibiting angiogenesis. There are many alkaloids, but this review takes into account only the most important ones. By considering the hypothetical amino acid precursors and origins, different plant-based alkaloids in use are sorted into different groups.  Table 2 summarizes the different alkaloids.

Table 3 summarizes studies that evaluated the effect of different alkaloids on angiogenesis in different types of disorders.

Almost all alkaloids show an antiproliferative and cytotoxic activity against cancer cell lines derived from several different histological origins (esophagus, stomach, colon, liver, lung, breast, bone, and brain), and this activity depends also on the activation of the expression of apoptotic genes [161]. On the other hand, *in vitro* studies showed that antiangiogenic effects rely on some shared ability to downregulate, in the same cancer cells, VEGF, TNF-*α*, and HIF-1*α* messengers and/or proteins levels (with mechanisms ranging from low expression and higher degradation), blocking the angiogenic cascade in endothelial cells, as shown for berberine [102, 106], noscapine [97], brucine [140], evodiamine [146], homoharringtonine [149], matrine [162], and tetrandrine [136]. For instance, studies have shown that the cascade is blocked by matrine and tetrandrine at the levels of STAT3 signaling ([163] and [164], respectively), while evodiamine inhibits *β*-catenin [165]. Similar results were reported for sanguinarine, capsaicin, taspine, harmine, and pterogynidine, for whose deeper dissection of the molecular pathways demonstrated that antiangiogenic activities specifically involve Akt phosphorylation [138, 166, 167], CDK expression [168], and NF-*κ*B translocation [160, 169] and that the effect is dose-dependent (10-300 nM) [166]. Other studies indicate that some alkaloids (sinomenine, brucine, and halofuginone) are able to directly regulate in vitro, at *μ*M concentrations, and in vivo in transplanted mice, the expression of angiogenic factors ([123, 140] and [170], respectively). In particular, sinomenine and halofuginone should be able to induce Smad protein depletion ([157, 171]).

Even if Ning et al. found that tetrandrine modulated, in human hypertrophic scar fibroblasts, the expression of miRNAs predicted to be related to wound healing [172], a process that is tightly related with angiogenesis [173], very little data is available at the moment on the role of miRNAs in alkaloid-associated angiogenic processes. One exception is for berberine that was shown to promote angiogenesis directly through miR-29b upregulation, upon induction of ischemia in mice [174]. The rest of the available data is restricted to experiments analyzing alkaloids as effectors of miR induction in different animal or cell models; for example, a recent study analyzed miRNA induction by berberine in porcine embryos [175]. Similarly, a berberine-containing commercially prepared traditional Chinese medicine Huang-Lian-Jiee-Dwu-Tang [176] was shown to induce VEGF and miR-126 expression in mesenchymal stem cell exosomes [177]. Interestingly, a recent paper suggests that berberine, together with evodiamine, is able to affect not only the expression of some miRs in colorectal cancer cells (in particular miR-29a) but also the expression of DNA methyltransferases that in turn regulate miRs' activities, providing evidence for epigenetic activities of alkaloids during carcinogenesis [178]. In other searches, the pulmonary arterial hypertension- (PAH-) inducing plant pyrrolizidine alkaloid monocrotaline was shown to affect miR-21 and let-7a expression in the lung of monocrotaline-injected rats [179]. The role of miR-21 in PAH was later confirmed in humans [180], but no data is available on miR-21's role on monocrotaline effects. Nonetheless, downregulation of miR-21, followed by the dephosphorylation of its target Akt, was reported also by the growth-inhibiting alkaloid matrine, on breast cancer cells [181]. Similar involvement of miR-21 by alkaloids was observed on human thyroid cancer cells [182], while other miRs were involved in other cells, i.e., miR-19b in melanoma [183], miR-106b in human acute T-cell lymphoblastic leukemia [184], and miR-126 in non-small-cell lung cancer [185].

Kaymaz et al. found that Capsaicin strongly reduced miR-520a expression in chronic myeloid leukemia cells [186]. As the regulation is associated with the inhibition of cell proliferation, the authors hypothesized that alkaloid's apoptotic properties could rely directly on miR-520a-5p/STAT3 interaction [186]. Similarly, data on rat hepatic stellate T6 cells suggest that the ability of sanguinarine to induce apoptosis through BCL2 downregulation could rely on the activation of miR-15a/16-1 expression [187]. On the other hand, homoharringtonine antileukemic effects were causatively related, by ectopic expression, to the activation of miR-370 and its target FoxM1, a major regulator in cell proliferation and apoptosis [188]. Other transfection experiments with miR mimics demonstrated unequivocally that inhibitory activities on invasion and metastasis by sinomenine on breast cancer cells depend on miR-324-5p and involve downstream NF-*κ*B [189].

## 6. Conclusion

Based on data reviewed here and their ability to act as regulatory agents, alkaloids from natural sources are promising molecules with an enormous potential for therapeutic intervention of many disorders. Compounds from natural sources are believed to be good candidates for the prevention of angiogenic disorders and also as complements for chemotherapy or radiotherapy, in combination with other anticancer therapies [50, 190–192]. Berberine, noscapine, sanguinarine, and taspine among others are the most interesting and promising angiogenesis inhibitors. Further investigations are still required to elucidate their precise molecular and cellular mechanisms, as well as any potential side effects.

## Figures and Tables

**Table 1:** Effect of different microRNAs on the angiogenic process.

MiRNA	Target gene	Role	Function	Reference
MiR-34a	Silent information regulator 1 (Sirt1)	MiR-34a has been found to target silent information regulator 1 (Sirt1), leading to cell cycle arrest or apoptosis	Antiangiogenesis	[67]
MiR-107	HIF-1*β*	MiR-107 decreases hypoxia signaling by suppressing expression HIF-1*β*	Antiangiogenesis	[75]
MiR-132	p120RasGAP	MiR-132 acts as an angiogenic switch by targeting p120RasGAP in the endothelium and thereby inducing neovascularization	Angiogenesis	[76]
MiR-424	Cullin 2 (CUL2)	MiR-424 targeted Cullin 2 (CUL2), a scaffolding protein critical to the assembly of the ubiquitin ligase system, thereby stabilizing HIF-*α* isoforms	Angiogenesis	[77]
MiR-93	Integrin-*β*8	MiR-93 promotes angiogenesis by suppressing integrin-*β*8 expression	Angiogenesis	[71]
MiR-29b	MMP-2	MiR-29b exerted its antiangiogenesis function, at least partly, by suppressing MMP-2 expression in tumor cells	Antiangiogenesis	[78]
MiR-519c	HIF-1*α*	Overexpression of miR-519c resulted in a significant decrease of HIF-1*α* protein levels and reduced the tube formation of human umbilical vein endothelial cells	Antiangiogenesis	[79]
MiR-210	VEGF and VEGFR	Overexpression of miR-210 enhances VEGF and VEGFR2 expression and promotes angiogenesis	Angiogenesis	[80]
MiR-155	Von Hippel-Lindau (VHL)	MiR-155 has a pivotal role in tumor angiogenesis by downregulation of VHL	Angiogenesis	[81]
MiR-195	VEGF, VAV2, CDC42	MiR-195 directly inhibited the expression of the proangiogenic factor VEGF and the prometastatic factors VAV2 and CDC42	Antiangiogenesis	[82]
MiR-145	*HIF-2α*	MiR-145 suppresses *HIF-2α* expression, thus inhibiting the angiogenesis	Antiangiogenesis	[83]
MiR-26a	HGF-hepatocyte growth factor receptor (cMet)	MiR-26a exerted its antiangiogenesis function, at least in part, by inhibiting HGF-hepatocyte growth factor (cMet) and its downstream signaling pathway	Antiangiogenesis	[84]
MiR-214	Hepatoma-derived growth factor (HDGF)	Downregulation of miR-214 contributes to the unusual hypervascularity of HCC via activation of the HDGF paracrine pathway for tumor angiogenesis	Antiangiogenesis	[85]
MiRNA-24	eNOS	Inhibition of microRNA-24 improves reparative angiogenesis in myocardial infarction	Antiangiogenesis	[86]
MiR-29a	Phosphatase and tensin homolog (PTEN)	TGF-*β*-regulated miRNA in promoting angiogenesis by targeting PTEN to stimulate AKT activity	Angiogenesis	[87]
MiR-27b	Vascular endothelial growth factor C (VEGFC)	MiRNA-27b targets vascular endothelial growth factor C to inhibit angiogenesis in colorectal cancer	Antiangiogenesis	[88]
MiR-503	FGF2 and VEGF-A	Demonstrate the antiangiogenesis role of miR-503 in tumorigenesis and provide a novel mechanism for hypoxia-induced FGF2 and VEGF-A through HIF1*α*-mediated inhibition of miR-503	Antiangiogenesis	[89]
MiR-143	Insulin-like growth factor-I receptor (IGF-IR)	Overexpression of miR-143 inhibited cell proliferation, migration, tumor growth, and angiogenesis and increased chemosensitivity to oxaliplatin treatment in an IGF-IR-dependent manner	Antiangiogenesis	[90]
MiR-382	Phosphatase and tensin homolog (PTEN)	MiR-382 induced by hypoxia promotes angiogenesis and acts as an angiogenic oncogene by repressing PTEN	Angiogenesis	[91]
MiR-210	Vascular endothelial growth factor (VEGF)	MiR-210 is a key factor at the microRNA level in promoting angiogenesis and neurogenesis, which was associated with local increased vascular endothelial growth factor (VEGF) levels	Angiogenesis	[92]
MiR-542-3p	Angiopoietin-2 (Angpt2)	MiR-542-3p inhibited translation of *Angpt2* mRNA by binding to its 3′ UTR, and the addition of miR-542-3p to cultured endothelial cells attenuated angiogenesis	Antiangiogenesis	[93]
MiR-214	Quaking	MiR-214 directly targets Quaking, a protein critical for vascular development. Quaking knockdown reduced proangiogenic growth factor expression and inhibited endothelial cell sprouting similar to miR-214 overexpression	Antiangiogenesis	[94]
MiR-20a	p300	P300 drives an angiogenic transcription program during hypertrophy that is fine-tuned in part through direct repression of p300 by miR-20a	Antiangiogenesis	[95]
MiR-15a	FGF2 and VEGF	*MiR-15a* negatively regulates angiogenesis *in vivo* and *in vitro* by suppression of FGF2 and VEGF	Antiangiogenesis	[96]

**Table 2:** Classification of different alkaloids based on amino acid precursors and their origins.

Amino acid precursors	Tyrosine	Tryptophan	Lysine
Alkaloid origins	*Berberine*	*Brucine*	*Matrine*
	Hydrastis canadensis; Coptis chinensis	Strychnos nux-vomica L.	Sophora alopecuroides
	*Noscapine*	*Evodiamine*	*Capsaicin*
	Papaver somniferum L.	Evodia rutaecarpa	Capsicum annum L.
	*Sanquinarine*	*Homoharringtonine*	*Halofuginone*
	Sanquinaria canadenis L.	Cephalotoxus	Dichroa febrifugus
	*Sinomenine*		*Pterogynidine*
	Sinomenium acutum		Pterogyne nitens Tul
	*Taspine*		
	*Tetrandarine*		
	**S**tephania tetrandra S.		

**Table 3:** Effects of different alkaloids on angiogenesis.

Name of alkaloid	*N*	Molecules		Biological results	Sample type	Year	Author
Noscapine	1	HIF-1	Decrease	Inhibit angiogenesis by downregulating VEFG	U87MG and T98G cell lines	2006	Newcomb et al. [97]
	2	VEGF	Decrease	Inhibit angiogenesis	Human T98 cell lines	2008	Newcomb et al. [98]
	3	VEGF	Decrease	Inhibit angiogenesis	Murine GL261 glioma cell lines	2008	Newcomb et al. [98]
	4	MMP-9 VEGF	Decrease	Inhibit angiogenesis	KBM-5, HL-60, Jurkat, HuT-78, U266, RPMI- 8226, H1299, A293, DU145, and SCC4 cell lines	2010	Sung et al. [99]
	5	VEGF	Decrease	Inhibit angiogenesis	The A549 or H460 cell	2010	Chougule et al. [100]
	6	VEGF	Decrease	Inhibit angiogenesis	Xenografted with H460 tumors (mice)	2010	Chougule et al. [100]
	7	HIF	Decrease	Inhibit angiogenesis	Ovarian cancer cells	2011	Su et al. [101]
	8	NFKB VEGF	Decrease	Inhibit angiogenesis	The human breast cancer cell lines MDA-MB-231 and MDA-MB-468	2011	Chougule et al. [100]

Berberine	9	HIF-1	Decrease	Inhibit angiogenesis by downregulating VEGF	Gastric adenocarcinoma cell line SC-M1	2004	Lin et al. [102]
	10	VEGF	Decrease	Inhibit angiogenesis	HeLa cell	2008	Lin et al. [103]
	11	VEGF MMP-2	Decrease	Inhibit angiogenesis	Human umbilical vein endothelial cells	2009	Gao et al. [104]
	12	Mmp-2/9 UPA	Decrease	Inhibit angiogenesis	Squamous cancer cell	2009	Ho et al. [105]
	13	VEGF	Decrease	Inhibit angiogenesis	Hepatocellular carcinoma	2010	C. Cheung et al.
	14	VEGF	Decrease	Inhibit angiogenesis	Hep G2 cell line	2011	Jie et al. [106]
	15	HIF-1 VEGF	Decrease	Inhibit angiogenesis	B16F-10 melanoma cells and C57BL/6 mice	2012	Hamsa and Kuttan [107]
	16	VEGF	Decrease	Inhibit angiogenesis	Xenografted hepatocellular carcinoma (mice)	2012	Ruhua [108]
	17	VEGF	Decrease	Inhibit angiogenesis	Hepatocellular carcinoma	2012	Wang et al.
	18	VEGF	Decrease	Inhibit angiogenesis	Breast cancer	2013	Kim et al. [109]
	19	VEGF	Decrease	Inhibit angiogenesis	HepG2 cells	2013	Wang and Ke [110]
	20	VEGF	Decrease	Inhibit angiogenesis	Human non-small-cell lung cancer	2013	Fu et al. [111]
	21	Mmp-2 UPA	Decrease	Inhibit angiogenesis	Cervical cancer	2014	Chu et al.[112]
	22	VEGF	Decrease	Inhibit angiogenesis	Hepatocellular carcinoma	2015	Tsang et al. [113]

Sanguinarine	23	VEGF	Decrease	Inhibit angiogenesis	Swine granulosa cell	2006	Bianco et al. [114]
	24	VEGF	Decrease	Inhibit angiogenesis	Endothelial cell line	2007	Basini et al. [115]
	25	VEGF	Decrease	Inhibit angiogenesis	Swine granulosa cells	2007	Basini et al. [116]
	26	VEGF	Decrease	Inhibit angiogenesis	Porcine aortic endothelial cell line	2007	Basini et al. [117]
	27	VEGF	Decrease	Inhibit angiogenesis	Swine ovarian follicles	2008	Basini et al. [116]
	28	?	?	Inhibit angiogenesis	B16 melanoma 4A5 cells	2009	De Stefano et al. [118]
	29	VEGF	Decrease	Inhibit angiogenesis	Human A549 lung cancer cells	2013	Xu et al. [119]
	30	VEGF	Decrease	Inhibit angiogenesis	MCF-7 human mammary adenocarcinoma cells	2013	Dong et al. [120]
	31	VEGF	Decrease	Inhibit angiogenesis	S180 sarcoma in mice	2014	Du et al. [121]
	32	HIF-1	Decrease	Inhibit angiogenesis	Pancreatic cancer	2015	Singh et al. [122]

Sinomenine	33	VEGF	Decrease	Inhibit angiogenesis	Human synovial sarcoma cells (Hs701.T)	2006	Li et al. [123]
	34	VEGF	Decrease	Inhibit angiogenesis	Umbilical vein endothelial cells (HUVEC) and U2OS cells	2016	Xie et al. [124]

Taspine	35	VEGF	Decrease	Inhibit angiogenesis	Chicken chorioallantoic membrane (CAM) neovascularization model and CAM transplantation tumor model	2008	Zhang et al. [125]
	36	VEGF	Decrease	Inhibit angiogenesis	Human umbilical vein endothelial cells	2008	Zhao et al. [126]
	37	VEGF	Decrease	Inhibit angiogenesis	Human umbilical vein endothelial cells	2010	Zhang et al. [127]
	38	VEGF	Decrease	Inhibit angiogenesis	Caco-2 cell lines	2011	Zhang et al. [128]
	39	VEGF	Decrease	Inhibit angiogenesis	Human liver cancer SMMC7721	2011	Zhang et al. [129]
	40	VEGF	Decrease	Inhibit angiogenesis	Chicken chorioallantoic membrane (CAM) and mouse colon tissue	2012	Zhang et al. [130]
	41	VEGF	Decrease	Inhibit angiogenesis	Human cell lines of SMMC-7721, A549, MCF-7, Lovo, and ECV304	2012	Zheng et al. [131]
	42	VEGF	Decrease	Inhibit angiogenesis	A549 cells lung cancer	2012	Lu et al. [132]

Tetrandrine	43	PDGF	Decrease	Inhibit angiogenesis	Adjuvant-induced chronic inflammation model of mouse	1998	Kobayashi et al. [133]
	44	VEGF	Decrease	Inhibit angiogenesis	RT-2 glioma RT	2009	Chen et al. [134]
	45	HIF-1 VEGF	Decrease	Inhibit angiogenesis	A rat model corneal neovascularization (CNV)	2011	Sun et al. [135]
	46	HIF-1 VEGF	Decrease	Inhibit angiogenesis	4T1 tumor bearing mice	2013	Gao et al. [136]
	47	—	—	Inhibit angiogenesis	Human umbilical vein endothelial cells (HUVECs) and the human colon cancer cell line Lovo	2013	Qian et al. [137]
	48	—	—	Inhibit angiogenesis	Liver cancer xenograft model in nude mice	2015	Xiao et al. [138]

Brucine	49	VEGF TGF-*β*	Decrease	Inhibit angiogenesis	Murine cannulated sponge implant angiogenesis model	2011	Agrawal et al. [139]
	50	VEGF	Decrease	Inhibit angiogenesis	Ehrlich ascites tumor and human cancer cell line	2011	Agrawal et al. [140]
	51	VEGF	Decrease	Inhibit angiogenesis	Nude mouse model of bone metastasis due to breast cancer	2012	Li et al. [141]
	52	HIF-1	Decrease	Migration and metastasis and angiogenesis	Hepatocellular carcinoma	2013	Shu et al. [142]
	53	VEGF	Decrease	Angiogenesis	Colon cancer cells	2013	Luo et al. [143]
	54	VEGF	Decrease	Inhibit angiogenesis	Lovo cell	2013	Zheng et al. [144]
	55	VEGF	Decrease	Inhibit angiogenesis	R breast cancer cell line MDA-MB-231	2013	Kechun and Zjauma [145]

Evodiamine	56	VEGF	Decrease	Inhibit angiogenesis	Human lung adenocarcinoma cell (CL1 cells)	2006	Shyu et al. [146]
	57	VEGF	Decrease	Inhibit angiogenesis	Breast cancer cell	2008	Wang et al. [147]
	58	HIF	Decrease	Inhibit angiogenesis	Human colon cancer cell	2015	Huang et al. [148]

Homoharringtonine	59	VEGF	Decrease	Inhibit angiogenesis	Human umbilical vein endothelial cell line (ECV304)	2004	Ye and Lin [149]
	60	VEGF	Decrease	Inhibit angiogenesis	Leukemic cell line (K562) in vitro	2004	Ye and Lin [149]
	61	VEGF	Decrease	Inhibit angiogenesis	Myeloid leukemia cells	2005	Fei and Zhang [150]
	62	HIF	Decrease	Inhibit angiogenesis	K562 cell	2008	Li et al. [151]

Matrine	63	VEGF	Decrease	Inhibit angiogenesis	Breast cancer cell	2009	Yu et al. [152]

Halofuginone	64	VEGF MMP	Decrease	Inhibit angiogenesis	BALB/c *ν*/*ν* (nude) mice	2003	Gross et al. [153]
	65	**—**	—	Inhibit angiogenesis	Metastatic rat brain tumor model	2004	Abramovitch et al. [154]
	66	TGF	Decrease	Inhibit angiogenesis	Polyoma middle T antigen mouse	2005	Yee et al. [155]
	67	TGF-*β*-1, bFGF, COL1A1, MMP-2, MMP-9, VEGF, and PDGF	Decrease	Inhibit angiogenesis	Xenografted (rat)	2012	Jordan and Zeplin [156]
	68	VEGF MMP TGF-*β*	Decrease	Inhibit angiogenesis	Acute promyelocytic leukemia mouse model	2015	Assis et al. [157]

Capsaicine	69	VEGF	Decrease	Inhibit angiogenesis	Endothelial cells in the rat aorta	2008	Pyun et al. [158]
	70	VEGF	Decrease	Inhibit angiogenesis	Non-small-cell lung cancer	2014	Chakraborty et al. [159]

Pterogynidine	71	—	—	Inhibit angiogenesis	Human umbilical vein endothelial cells (HUVEC)	2009	Lopes et al. [160]
